# 1065. Patient-Reported Reasons for Non-Participation in Inpatient COVID-19 Clinical Research: Results from a Multi-Center VA Embedded Randomized Trial

**DOI:** 10.1093/ofid/ofac492.906

**Published:** 2022-12-15

**Authors:** Samira Reyes Dassum, Ryan Ferguson, Patricia Woods, Maura Flynn, Karen Visnaw, Sara J Schiller, Paul Monach, Sarah Leatherman, Westyn Branch-Elliman

**Affiliations:** Roger Williams Medical Center, Providence, Rhode Island; VA Boston Healthcare System, Boston, Massachusetts; VA Boston Healthcare System, Boston, Massachusetts; VA Boston Healthcare System, Boston, Massachusetts; VA Boston Healthcare System, Boston, Massachusetts; VA Boston Healthcare System, Boston, Massachusetts; VA Boston Healthcare System, Boston, Massachusetts; VA Boston Healthcare System, Boston, Massachusetts; VA Boston Healthcare System, Boston, Massachusetts

## Abstract

**Background:**

Early in the COVID-19 pandemic, no evidence-proven therapeutics were approved, and thus participation in a clinical trial was often the only way to access experimental medications. However, in the US, participation in medical research is low. Patient-stated factors impacting enrollment decisions are poorly characterized. Thus, the aim of this study was to identify patient-reported reasons for declining enrollment in a COVID-19 clinical trial.

**Figure:**
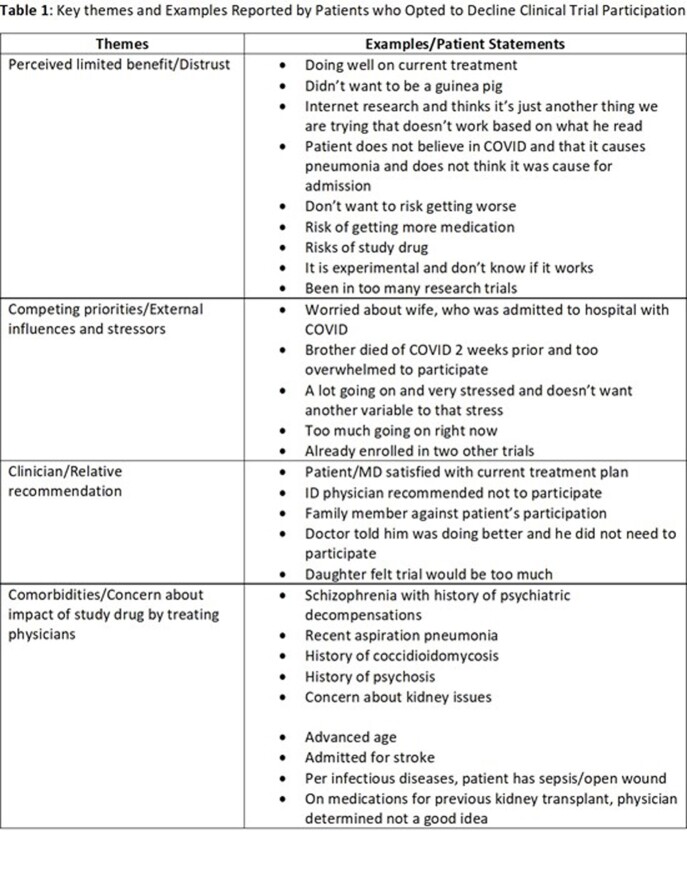

**Methods:**

As part of an open-label, pragmatic randomized trial across five VAs from 4/10/20 – 2/3/21, SARS-COV-2 positive inpatients with moderate to severe disease were screened for eligibility. Potentially eligible patients received an informed consent packet with a letter explaining the study and were then contacted remotely to assess willingness to participate. If eligible patients declined to participate, if willing, they were asked an open-ended question about the reasons behind their decision. If they were not able to provide a specific answer, then general categories were offered. Qualitative responses were analyzed using a directed content analysis approach; responses were categorized using previously defined factors that have contributed to decisions not to enroll in other clinical therapeutics trials, primarily conducted in oncology.

**Results:**

Among 417 patients screened, 162 met eligibility criteria. Of these, 53 consented (32.7%), 102 declined to participate (62.9%), and 7 were unable to give consent. Themes and examples of reasons for declining participation are presented in Table 1. Among the patients who declined to participate, the four most cited factors were limited perceived benefit, competing priorities, physician or family influence and presence of comorbidities. Several patients reported that their decision was influenced by physician or family recommendation to decline, which was reported as physician lack of support for participation due to the presence of comorbidities or physician perceived lack of benefit given clinical improvement prior to study enrollment.

**Conclusion:**

Understanding reasons and attitudes driving enrollment may help investigators address them during the recruitment process and increase participation in clinical trials in the US.

**Disclosures:**

**Karen Visnaw, RN**, Hologic Inc: Stocks/Bonds|Liquidia Corporation: Stocks/Bonds **Paul Monach, MD, PhD**, Celgene / Bristol-Myers Squibb: Advisor/Consultant|ChemoCentryx: Advisor/Consultant|Gilead: Grant/Research Support|Kiniksa: Advisor/Consultant **Westyn Branch-Elliman, MD, MMSc**, DLA Piper,LLC/Medtronic: Advisor/Consultant|Gilead Pharmaceuticals: Grant/Research Support.

